# Granulocyte Transfusions in Neutropenic Infections: Insights From a Single-Center Study

**DOI:** 10.7759/cureus.55953

**Published:** 2024-03-11

**Authors:** Sidika Gülkan Özkan, Ali Kimiaei, Seyedehtina Safaei, Meral Sönmezoğlu, Hasan Atilla Özkan

**Affiliations:** 1 Hematology, Bahçeşehir University, Istanbul, TUR; 2 Infectious Diseases, Yeditepe University Hospital, Istanbul, TUR

**Keywords:** granulocyte transfusion, neutropenic infections, infection control, febrile neutropenia, neutropenia

## Abstract

Introduction

Despite the development of modern antibiotic and antifungal therapies, neutropenic infections remain life-threatening. Granulocyte transfusion (GTX) is a less frequently used treatment modality in patients with refractory neutropenic infections. The role of donor GTX remains controversial, partly because of the lack of proper clinical trials. This study aimed to contribute to the literature by evaluating the efficacy and side effects of granulocyte transfusions in our center.

Methods

Eight febrile neutropenic patients with confirmed infections received granulocyte transfusions from ABO-compatible related and unrelated donors. Donors received filgrastim and dexamethasone stimulation, and granulocyte suspensions were irradiated and administered within six hours. Monitoring, antibiotic therapy, and granulocyte colony-stimulating factor (G-CSF) support were maintained.

Results

Our study observed a 28-day survival rate of 25%, which was lower than that reported in previous literature. The median number of transfusions was 3, with an average eight-day duration post-infection diagnosis, and no side effects were observed.

Conclusion

While some patients benefited from GTX, overall survival rates remained modest, indicating the need for further research. Prospective, well-powered randomized controlled trials are essential to address patient selection, dosing, and duration to determine the clinical utility of GTX. This study underscores the complexity of GTX in real-world clinical practice and provides insight into the ongoing debate regarding its efficacy in treating severe neutropenic infections.

## Introduction

Granulocytes are versatile cells that contribute to both innate and adaptive immunity, playing a crucial role in host defense against pathogens [1]. Neutropenia is a condition characterized by a low level of neutrophils. Studies conducted many years ago [2] have demonstrated the relationship between the number of circulating neutrophils and infections. Granulocyte transfusion (GTX) has been administered to patients with granulocytopenia and antibiotic-resistant infections, with varying outcomes depending on factors such as the extent and duration of bone marrow insufficiency, type and grade of infection, age, platelet deficiency, and liver and renal insufficiency [3]. Although GTX remains a treatment modality for refractory neutropenic infections, its efficacy remains unclear [4]. Patient selection, adequate dosing, and endpoint evaluation are significant challenges in this treatment approach [5]. In general, indications for GTX can be divided into (i) generally accepted indications that include documented severe bacterial infection unresponsive to 24-48 hours of appropriate antibiotic therapy in a patient with severe neutropenia and neutrophil dysfunction, and (ii) less clear indications that include documented severe fungal infection unresponsive to appropriate antifungal therapy in a patient with severe neutropenia [6]. In this article, we report a retrospective analysis of GTX practice in our institution for treating febrile neutropenic patients.

## Materials and methods

Study design, patient selection, and donor eligibility

We conducted a retrospective review of the data of eight patients who underwent granulocyte transfusions between 2018 and 2020, along with their respective donors. Patients who (i) were over 18 years of age, (ii) had febrile neutropenia independent of underlying disease, and (iii) received granulocyte infusions between 2018 and 2020 were included in the study. In our center, the procedure for granulocyte collection was standardized according to data in the literature [7]. Eligible donors, comprising both ABO-compatible volunteer-related and unrelated individuals, were stimulated with subcutaneous filgrastim (0.5 mcg/kg) and oral dexamethasone (8 mg).

Granulocyte collection and processing

Granulocyte suspensions were collected through a continuous flow centrifugation method using the TerumaBCT Optia device. These suspensions were irradiated with 25 Gy of cesium at the 12-hour mark.

Infusion procedure

Neutropenia was classified by the absolute neutrophil count, with mild defined as 1.0-1.5 × 109/L, moderate as 0.5-0.9 × 10^9^/L, and severe as less than 0.5 × 10^9^/L [8]. Granulocyte suspensions were administered within six hours to febrile neutropenic patients with confirmed infections. As a premedication regimen, diphenhydramine and acetaminophen were administered 30 minutes before the infusion.

Monitoring and follow-up

After the infusion, leukocyte and neutrophil counts were monitored at the 12-hour mark, and daily granulocyte infusions were maintained as long as donors were available. All patients continued to receive appropriate antibiotic and antifungal therapies, coupled with granulocyte colony-stimulating factor (G-CSF) support. A minimum interval of four hours was observed between the granulocyte infusion and amphotericin B treatment, and treatment ceased when infections were under control, neutrophil counts exceeded 1000/mm3, or in the unfortunate event of patient loss.

Statistical analysis

Due to the low number of patients, statistical analysis was not conducted. The purpose of this study is to share the results of granulocyte transfusions at our center.

## Results

Patient characteristics

Eight patients were included in the study, with a median age of 56.6 years (range: 22-78). Among them, five were female, and three were male. The diagnoses included acute myeloid leukemia (AML) in four patients (50%), acute lymphoblastic leukemia (ALL) in two patients (25%), haploidentical transplantation in one patient (12.5%), and recurrent gastric cancer in one patient (12.5%).

Granulocyte transfusion details

The median number of granulocyte transfusions administered per patient was 3 (range: 1-6). Three patients received a granulocyte transfusion once, two patients received it six times, two patients received it three times, and one patient received it four times. The mean granulocyte transfusion volume was 2.72 × 10^10^ cells (range: 1.71-4.46 × 10^10^). Granulocyte transfusions were administered within an average of eight days (range: 3-18). Table 1 presents patient information and treatment details.

**Table 1:** Patient information and treatment details. Hyper-CVAD regimen: cyclophosphamide, vincristine sulfate, doxorubicin hydrochloride (Adriamycin), methotrexate, cytarabine, and dexamethasone; R-CODOX-M regimen: rituximab, cyclophosphamide, vincristine, doxorubicin, and methotrexate; Flu/BU/ATG regimen: fludarabine, busulfan, and anti-thymocyte globulin.

Patient	Age (years)	Diagnosis	Treatment regimen	History of stem cell transplantation	Day of administration	Number of administrations	Product (10^10^ cells)
1	74	AML	Azacitidine	No	8	1	2.23
2	22	B-ALL	Hyper-CVAD	No	16	3	3.38
3	66	B-ALL	R-CODOX-M	No	5	6	2.11
4	78	AML	Azacitidine, venetoclax	No	7	6	2.24
5	22	AML	Idarubicin, cytarabine	No	4	1	4.46
6	58	AML	Idarubicin, cytarabine	No	3	4	3.29
7	67	Recurrent gastric cancer	Irinotecan, Capecitabine	No	3	3	2.35
8	65	Haploidentical SCT	Flu/Bu/ATG	Yes	18	1	1.71

Infection details and clinical outcomes

Three patients (37.5%) had pneumonia, three patients (37.5%) had Gram-negative sepsis, one patient (12.5%) had an invasive fungal infection, and one patient (12.5%) had a catheter-related infection. Broad-spectrum antibiotics such as meropenem and amikacin were commonly used to treat bacterial infections, while antifungal agents such as amphotericin B were employed to manage fungal infections. The choice of antimicrobial therapy was guided by the underlying infection source and the patient's clinical status. No side effects were observed, and the 28-day survival rate was 25%. Infection details and clinical outcomes are summarized in Table 2.

**Table 2:** Infection details and clinical outcomes.

Patient	Source of infection	Presence of sepsis	Intensive care monitoring	Ventilator support	Antibiotic regimen	Antifungal regimen	28-day survival
1	Pneumonia	Yes	Yes	Yes	Meropenem, Teicoplanin, Levofloxacin, and Amikacin	Amphotericin B	Dead
2	Invasive fungal infection	Yes	Yes	No	Meropenem, Teicoplanin, and Amikacin	Amphotericin B	Dead
3	Catheter infection	Yes	Yes	No	Meropenem, Amikacin, and Teicoplanin	Amphotericin B	Dead
4	Pneumonia	Yes	Yes	Yes	Meropenem, Teicoplanin, Gentamicin, and TMP-SMX	Amphotericin B	Dead
5	Gram-negative sepsis	Yes	No	No	Meropenem, Amikacin, and Teicoplanin	Amphotericin B	Alive
6	Gram-negative sepsis	Yes	Yes	No	Meropenem, Amikacin, and Teicoplanin	Amphotericin B	Dead
7	Gram-negative sepsis	Yes	Yes	No	Piperacillin/tazobactam and Ciprofloxacin	Amphotericin B	Alive
8	Pneumonia	Yes	Yes	Yes	Meropenem and Teicoplanin	Amphotericin B	Dead

## Discussion

In this study, we investigated the effect of granulocyte transfusion on infection control in different patient groups with severe febrile neutropenia. There were no specific side effects, and the 28-day survival rate was 25%.

Before the 1990s, the results of case series and controlled trials of GTX in adult patients spanned from evident to modest or absent benefits, causing a decrease in the popularity of GTX [9-21]. While some early studies have shown efficacy [9,11,13], others were all negative, with high success rates in the control groups [15,21,22].

The prevailing theory points to the inadequate dosage of granulocytes as the root cause of the ineffectiveness. After the approval of G-CSF by the FDA in 1991, higher yields of granulocytes were achieved, leading to renewed interest in GTX [7]. The addition of dexamethasone to G-CSF further increased the yield [23]. Since then, many centers have used a combination of G-CSF and dexamethasone; however, this approach is not universal [24].

In the post-G-CSF era, many studies have tried to answer the question of whether the use of GTX is clinically beneficial. The mixed results of recent studies on the survival advantages of GTX once again failed to confirm or disprove the benefits of GTX. While patients in some studies experienced clear clinical benefits when compared to historical controls [25], situations with no notable variance in mortality rates [26] and even cases where patients with fungal infections who underwent GTX had a heightened risk of mortality compared to those who did not have been reported [27]. 

Recent studies have suggested that the efficacy of granulocyte transfusions in neutropenic patients is proportional to the dose of transfused granulocytes. Doses of at least 1 × 10^10^ granulocytes per transfusion appear to be required to treat or prevent infections [28,29], and if there is a benefit from giving granulocytes, it is likely derived from higher doses, for example, above 0.6 × 10^9^/kg per dose [30,31].

In a phase III randomized control trial by Seidel et al. [32], they included 74 patients with hematological malignancies who subsequently underwent GTX and found that the probability of 28-day survival after randomization was >80% in both groups, and no effect of GTX on survival until day 100 could be detected in patients with fungal, bacterial, or unknown infection and various levels of neutropenia. Furthermore, the study conducted by Price et al. [31] is the largest randomized controlled trial of GTX in 114 patients. They investigated the efficacy of transfusion with granulocytes from G-CSF/dexamethasone-treated donors in neutropenic patients with a neutrophil count <0.5 × 10^9^/L and a proven or probable bacterial or fungal infection. The principal outcome measure was a composite of survival and microbial responses evaluated 42 days after randomization. With 56 subjects in the granulocyte arm and 58 in the control arm, individuals in the granulocyte group received a median of five transfusions with a mean dose of 54.9 × 10^9^ granulocytes. The overall success rate was 42% for the granulocyte group and 43% for the control group, with no significant difference between the two groups. Interestingly, the success rates did not differ between the granulocyte and control arms across different types of infections. A post-hoc analysis suggested that individuals who received an average dose of at least 0.63 × 10^9^ granulocytes per kilogram tended to have better outcomes. Overall, these studies found no effect of granulocyte transfusion on the primary outcome, likely due to their lower enrollment than planned and being underpowered, which limits their ability to detect a potentially beneficial effect.

In contrast to these findings, GTX efficacy has been demonstrated in single-center studies with a limited number of patients. In a study by Al-Tanbal et al., it was shown that GTX has a beneficial effect when administered to febrile neutropenic patients with hematological disorders during various phases of treatment, including chemotherapy and bone marrow transplantation [33]. They found a response rate of approximately 60%, similar to that reported in other studies [6,34,35]. For example, Ofran et al. showed a clinical efficacy of 64.3% in a cohort of 47 patients with hematological disorders who had severe neutropenia with documented infection and a mortality rate of 38% [35].

In our study, the mortality rate was 75%, which was quite high. This result may be related to the heterogeneous group of patients and the lack of standardization of the day of neutropenia on which GTX transfusion was administered.

This study has some limitations, including the small sample size of eight patients, the retrospective design, and the absence of a control group for outcome comparisons. Uncontrolled factors, such as variations in bone marrow insufficiency, infection specifics, age, platelet deficiency, and organ insufficiency, may have influenced the results. In addition, the amount of granulocytes applied is not above 0.63 × 10^9^ per kilogram of patient per administration.

## Conclusions

In conclusion, this study provides some evidence to support the lack of beneficial effects of GTX in the treatment of neutropenic patients with severe infections. Well-controlled prospective studies of GTX efficacy and inefficacy are needed to clarify transfusion timing, dose, therapeutic indications, and the maximum duration of treatment.

## References

[1] Minton K (2011). Granulocytes: a weighty role for eosinophils. Nat Rev Immunol.

[2] Bodey GP, Buckley M, Sathe YS, Freireich EJ (1966). Quantitative relationships between circulating leukocytes and infection in patients with acute leukemia. Ann Intern Med.

[3] Bishton M, Chopra R (2004). The role of granulocyte transfusions in neutropenic patients. Br J Haematol.

[4] Cohen T, Simmons SC, Pham HP, Staley EM (2021). Granulocyte transfusion: clinical updates and a practical approach to transfusion. Clin Lab Med.

[5] Creasey T, Jones GL, Collin M (2016). Granulocyte infusion: benefit beyond neutrophils?. Transfus Med.

[6] Price TH, Bowden RA, Boeckh M, Bux J, Nelson K, Liles WC, Dale DC (2000). Phase I/II trial of neutrophil transfusions from donors stimulated with G-CSF and dexamethasone for treatment of patients with infections in hematopoietic stem cell transplantation. Blood.

[7] Bensinger WI, Price TH, Dale DC (1993). The effects of daily recombinant human granulocyte colony-stimulating factor administration on normal granulocyte donors undergoing leukapheresis. Blood.

[8] Hay D, Hill M, Littlewood T (2014). Neutropenia in primary care. BMJ.

[9] Higby DJ, Yates JW, Henderson ES, Holland JF (1975). Filtration leukapheresis for granulocyte transfusion therapy. Clinical and laboratory studies. N Engl J Med.

[10] Alavi JB, Root RK, Djerassi I (1977). A randomized clinical trial of granulocyte transfusions for infection in acute leukemia. N Engl J Med.

[11] Herzig RH, Herzig GP, Graw RG Jr, Bull MI, Ray KK (1977). Successful granulocyte transfusion therapy for gram-negative septicemia. A prospectively randomized controlled study. N Engl J Med.

[12] (1977). Protocol for a cooperative trial of empirical antibiotic treatment and early granulocyte transfusions in febrile neutropenic patients. Eur J Cancer (1965).

[13] Vogler WR, Winton EF (1977). A controlled study of the efficacy of granulocyte transfusions in patients with neutropenia. Am J Med.

[14] Clift RA, Sanders JE, Thomas ED, Williams B, Buckner CD (1978). Granulocyte transfusions for the prevention of infection in patients receiving bone-marrow transplants. N Engl J Med.

[15] Scali G, von Felten A, Fehr J, Sauter C, Gmur J (1978). [Granulocyte substitution in febrile leukemia patients with bone marrow aplasia. 1. Results of a prospective study]. Schweiz Med Wochenschr.

[16] Mannoni P, Rodet M, Vernant JP (1979). Efficiency of prophylactic granulocyte transfusions in preventing infections in acute leukaemia. Rev Fr Transfus Immunohematol.

[17] Winston DJ, Ho WG, Young LS, Gale RP (1980). Prophylactic granulocyte transfusions during human bone marrow transplantation. Am J Med.

[18] Strauss RG, Connett JE, Gale RP (1981). A controlled trial of prophylactic granulocyte transfusions during initial induction chemotherapy for acute myelogenous leukemia. N Engl J Med.

[19] Ford JM, Cullen MH, Roberts MM, Brown LM, Oliver RT, Lister TA (1982). Prophylactic granulocyte transfusions: results of a randomized controlled trial in patients with acute myelogenous leukemia. Transfusion.

[20] Winston DJ, Ho WG, Gale RP (1982). Therapeutic granulocyte transfusions for documented infections. A controlled trial in ninety-five infectious granulocytopenic episodes. Ann Intern Med.

[21] Bow EJ, Schroeder ML, Louie TJ (1984). Pulmonary complications in patients receiving granulocyte transfusions and amphotericin B. Can Med Assoc J.

[22] Klastersky J, Zinner SH, Gaya H (1983). Early granulocyte transfusions in high risk febrile neutropenic patients. Schweiz Med Wochenschr Suppl.

[23] Stroncek DF, Yau YY, Oblitas J, Leitman SF (2001). Administration of G--CSF plus dexamethasone produces greater granulocyte concentrate yields while causing no more donor toxicity than G--CSF alone. Transfusion.

[24] Strauss RG, Klein HG, Leitman SF (2011). Preparation of granulocyte concentrates by apheresis: collection modalities in the USA. Vox Sang.

[25] Rutella S, Pierelli L, Sica S (2003). Efficacy of granulocyte transfusions for neutropenia-related infections: retrospective analysis of predictive factors. Cytotherapy.

[26] Safdar A, Hanna HA, Boktour M (2004). Impact of high-dose granulocyte transfusions in patients with cancer with candidemia: retrospective case-control analysis of 491 episodes of Candida species bloodstream infections. Cancer.

[27] Raad II, Chaftari AM, Al Shuaibi MM (2013). Granulocyte transfusions in hematologic malignancy patients with invasive pulmonary aspergillosis: outcomes and complications. Ann Oncol.

[28] Estcourt LJ, Stanworth S, Doree C, Blanco P, Hopewell S, Trivella M, Massey E (2015). Granulocyte transfusions for preventing infections in people with neutropenia or neutrophil dysfunction. Cochrane Database Syst Rev.

[29] Estcourt LJ, Stanworth SJ, Hopewell S, Doree C, Trivella M, Massey E (2016). Granulocyte transfusions for treating infections in people with neutropenia or neutrophil dysfunction. Cochrane Database Syst Rev.

[30] Elebute MT, Massey EJ, Benjamin S, Stanworth SJ (2012). Clinical guidelines for the use of granulocyte transfusions. Semantic Scholar.

[31] Price TH, Boeckh M, Harrison RW (2015). Efficacy of transfusion with granulocytes from G-CSF/dexamethasone-treated donors in neutropenic patients with infection. Blood.

[32] Seidel MG, Peters C, Wacker A (2008). Randomized phase III study of granulocyte transfusions in neutropenic patients. Bone Marrow Transplant.

[33] Al-Tanbal H, Al Humaidan H, Al-Nounou R, Roberts G, Tesfamichael K, Owaidah T (2010). The value and practicality of granulocyte transfusion: a single oncology centre experience. Transfus Med.

[34] Cesaro S, Chinello P, De Silvestro G (2003). Granulocyte transfusions from G-CSF-stimulated donors for the treatment of severe infections in neutropenic pediatric patients with onco-hematological diseases. Support Care Cancer.

[35] Ofran Y, Avivi I, Oliven A (2007). Granulocyte transfusions for neutropenic patients with life-threatening infections: a single centre experience in 47 patients, who received 348 granulocyte transfusions. Vox Sang.

